# Embryo-fetal and early-life protein restriction programs pulmonary structure and molecular parameters in both dams and female offspring

**DOI:** 10.1007/s11010-026-05653-4

**Published:** 2026-07-24

**Authors:** Flávia Alessandra Maciel, Matheus Naia Fioretto, Luisa Annibal Barata, Isabelle Tenori Ribeiro, Vinícius Alexandre de Andrade Felipe, Lucas Sobrinho Lemos, Maycon Tavares Emílio-Silva, Tainá Dorte da Silva, Flávia Karina Delella, Clélia Akiko Hiruma Lima, Wellerson Rodrigo Scarano, Luis Antonio Justulin

**Affiliations:** 1 Department of Cellular and Molecular Biology, Institute of Biosciences, Sao Paulo State University, Botucatu, SP Brazil; 2 Brazilian National Institute of Science and Technology in DOHaD, Botucatu, Brazil; 3 Department of Physiology, Institute of Biosciences, Sao Paulo State University, Botucatu, SP 18618-689 Brazil

**Keywords:** DOHaD, Early Life, Lung, Maternal health, Maternal malnutrition

## Abstract

The Developmental Origins of Health and Disease (DOHaD) theory proposes that adverse conditions during critical developmental windows can increase disease susceptibility throughout life. Maternal protein restriction (MPR) is a well-established experimental model that impairs offspring lung development, affecting pulmonary structure, metabolism, and molecular pathways from early life to aging. However, little is known about its effects on maternal pulmonary health and female offspring, representing an important gap in the literature. Therefore, this study investigated the effects of MPR on lung structure and metabolism in dams at the end of lactation and in female offspring at weaning. *Sprague Dawley* rats were fed either a control diet (17% protein) or a low-protein diet (6% protein) throughout gestation and lactation. Dams and female offspring were euthanized at weaning, and pulmonary alterations were assessed through histological, biochemical, molecular, zymographic, and in silico analyses. MPR increased collagen deposition, mast cell density, and *Acta* expression, while reducing reticular fibers in maternal lungs, accompanied by a trend toward increased lipid peroxidation. In female offspring, MPR reduced alveolar diameter, mast cell density, and reticular fibers, while increasing collagen deposition, MMP-2 activity, PI3K and *MyoD* expression, and decreasing mTOR expression as well as *Nduf* transcript levels. No significant alterations in antioxidant parameters were observed in either dams or offspring. Integrative analyses indicate that MPR promotes structural remodeling in maternal lungs, potentially compromising ventilatory function. In female offspring, MPR induces delayed pulmonary development, extracellular matrix remodeling, alterations in smooth muscle-related pathways, and disturbances in energy metabolism. Together, these findings demonstrate that maternal protein resQ1triction adversely affects pulmonary health in both dams and female offspring, revealing potential mechanisms linking early-life nutritional adversity to the developmental origins of respiratory disease.

**Supplementary Information:**

The online version contains supplementary material available at https://doi.org/10.1007/s11010-026-05653-4.

## Introduction

In 2022, a staggering 149 million children under the age of 5 were classified as stunted, and 45 million were identified as wasted. There has been a growing medical-scientific interest in investigating the Developmental Origins of Health and Disease (DOHaD) [1–5]. Alarmingly, almost half of all deaths among children under the age of 5 are attributed to undernutrition, which is predominantly prevalent in low- and middle-income countries. It plays a role in different acute and chronic disease conditions [6, 7], including respiratory diseases and lung development [8, 9]. Furthermore, there are many windows of vulnerability, including breastfeeding, early childhood, and adolescence, all of which are essential developmental stages [10–13].

Maternal protein restriction (MPR), which affects approximately 8% of the global population, is a significant developmental challenge that can impair multiple physiological systems in the offspring, resulting in low birth weight and restricted fetal growth [14–16]. Beginning in utero, MPR may adversely affect lung development both directly, by reducing alveolar formation, and indirectly, through conditions such as intrauterine growth restriction, prematurity, and low birth weight [17]. Experimental studies have demonstrated that early-life malnutrition is associated with pulmonary injury and impaired lung development, including reduced pulmonary protein and DNA synthesis [18]. Conversely, adequate postnatal nutrition and breastfeeding appear to exert protective effects. Overall, malnutrition can compromise respiratory health and lung function through multiple structural, cellular, and metabolic mechanisms.

Early life lung development lays the basis for homeostatic lung growth, function, and adaptations to the environment throughout the lifespan. Deregulation in these tightly regulated processes has been related to the developmental origins of many lung diseases [19, 20]. Like the kidney and prostate, the lung develops and grows through a complex mechanism named branching morphogenesis [21]. The lung has numerous roles, ranging from hematosis to connections with the cardiovascular and endocrine systems, and is one of the essential organs that can be damaged early in life with long-term repercussions during development [18, 22]. Although the number of alveoli, alveolar ducts, and capillary segments remains constant in maturity, alveolar size and alveolar-capillary surface area rise significantly with age [23]. Multiple structural and functional changes in the respiratory system are linked with normal lung aging [23, 24]. Longitudinal studies have shown that impaired pulmonary function is a predictor of morbidity and death and may encourage the development of numerous disease processes [25, 26].

Within the DOHaD framework, the primary focus has traditionally been on understanding how adverse developmental environments influence offspring health trajectories across the lifespan. However, the consequences of developmental insults on female offspring remain incompletely understood, highlighting an important knowledge gap that the present study addresses. Moreover, DOHaD research has historically placed less emphasis on maternal health outcomes, despite the likelihood that MPR imposes significant biological burdens during pregnancy, lactation, and potentially later life. Expanding this perspective is essential to integrate maternal health into the broader developmental programming paradigm. Pregnancy and lactation are characterized by profound neuroendocrine, metabolic, and physiological adaptations aimed at supporting fetal growth and postnatal development. Under conditions of nutritional deprivation, these adaptive mechanisms may prioritize offspring survival at the expense of maternal tissue homeostasis. In this context, pulmonary structure and function may be particularly vulnerable, potentially compromising gas exchange capacity and triggering compensatory responses that extend beyond the respiratory system to influence cardiovascular and systemic physiology. Although such adaptations may initially serve protective functions, they could also contribute to long-term maternal physiological alterations.

Importantly, maternal responses to nutritional stress may have implications that extend beyond maternal health. Alterations in maternal phenotype can directly influence placental function and fetal development during gestation, while also affecting the composition and biological quality of breast milk during lactation. Consequently, maternal pulmonary and systemic adaptations should be viewed not only as outcomes of developmental programming but also as potential determinants of offspring developmental trajectories. From this broader perspective, the present findings reinforce the importance of investigating the lungs within a dual framework that encompasses both DOHaD principles and women’s health, recognizing the mother as both a target and a mediator of developmental programming processes.

In males, we previously observed that MPR led to delayed lung development early in life, along with structural and molecular alterations associated with immune responses and cellular stress during aging [27]. However, regarding sex-specific differences, the literature remains limited in addressing the effects of MPR on female lungs, and even fewer studies have examined its direct impact on maternal lung structure. Therefore, the aim of this study was to analyze how MPR affects maternal and female lungs at postnatal day (PND) 21, with emphasis on morphological and molecular parameters. We hypothesized that MPR alters maternal lung structure—potentially associated with ventilatory changes—and also induces early alterations in female offspring, particularly in pulmonary metabolic and structural parameters.

## Materials and methods

### Animals and experimental design

Adult female (90 days old, *n* = 40) and male (90 days old, *n* = 20) *Sprague Dawley* rats were acquired from the Central Animal Facility of the State University of Campinas (Campinas, SP, Brazil). The animals were kept in a controlled environment with a temperature between 22 and 25 °C, relative humidity of 55%, and a 12-hour photoperiod, in addition to free access to filtered water and feed. All procedures followed the ethical principles of animal research and established Brazilian legislation and were authorized by the Animal Experimentation Ethics Committee of the Institute of Biosciences/UNESP (CEUA No. 7376171224). Throughout the experiment, the housing and use of the animals were carried out in accordance with the appropriate guidelines and regulations. Efforts were made to minimize suffering and reduce the number of animals used in the experiments. Data acquisition and description followed the recommendations established in the ARRIVE guidelines.

Mating occurred at night in a harem system (1 male to 3 females). After confirmation of pregnancy (Gestational Day 1 - GD1) by detection of spermatozoa in vaginal smear, pregnant rats were divided into two experimental groups: (1) Control group (CTR, *n* = 20), which received a standard diet (17% protein) during gestation and lactation, and (2) Low-protein diet group during gestation and lactation (GLLP, *n* = 20), which received a low-protein diet (6% protein) during gestation and lactation. The diets were isocaloric and normosodic and were provided by PragSoluções (PragSoluções, SP, Brazil) [28]. The nutritional information was presented in Naia Fioretto et al., 2024 [27]. The experimental design is represented in the Supplementary Fig. 1.

At PND21, post-lactation dams and their offsprings were weighed, anesthetized, and euthanized (*n* = 5/group). The lungs were collected and weighed, immersed in fixative, and processed using the histological techniques (*n* = 5/group) described below, and another was immediately frozen in liquid nitrogen (*n* = 5/group) and kept in a freezer at -80 °C until used.

### Histological analyses

Lung samples from post-lactation dams and their female offspring at PND21 (*n* = 5/group) were fixed in metacarn (70% methanol + 20% chloroform + 10% acetic acid) [29], dehydrated in ethanol, cleared in xylene, and embedded in Paraplast (Sigma Co, Saint Louis, MO). Histological Section (5 μm) were collected on silanized slides and processed for morphological analyses.

The histological parameters were based on Naia Fioretto et al. 2024 [27]. Briefly, lung paraplast Sections (5 μm) from all experimental groups were processed for morphological analysis using hematoxylin and eosin [30], picrosirius red for type I and III collagen fibers [31], 1% toluidine blue (pH 2.4) for mast cell quantification [32], and resorcinol–fuchsin (Weigert) or reticulin staining for elastic and reticular fibers, respectively. Morphometric analyses included mean alveolar septal thickness (50 random measurements at 400×), mean alveolar diameter (50 measurements at 200×), collagen fiber area (automatic detection at 200×), mast cell density (counts at 400×), and total area of elastic and reticular fibers (automatic detection at 40×), all evaluated across multiple microscopic fields from independent lung sections. Histological images were obtained using a Leica DMLB microscope coupled to a Leica DC300FX digital camera, and analyses were performed with Leica Q-Win software (version 3.0 for Windows).

### Protein extraction and zymography

The complete gelatin zymography protocol was described by Justulin et al. (2010) [33]. Briefly, frozen lung samples from post-lactation dams and their female offspring at PND21 (*n* = 5/group) were mechanically homogenized in extraction buffer using a Polytron homogenizer. Protein concentration was determined by the Bradford method [34]. Equal amounts of protein (35 µg) from each sample were subjected to electrophoresis.

Gels were then incubated for 20 h at 37 °C in 50 mM Tris–HCl buffer (pH 8.4) containing 5 mM CaCl₂ and subsequently stained with Coomassie Brilliant Blue R-250 (Sigma-Aldrich, St. Louis, MO, USA). Gelatinolytic activities of MMP-2 and MMP-9 were identified as clear bands, which were scanned and quantified by densitometry. Band intensity was measured as integrated optical density (IOD) using ImageJ software (National Institutes of Health, USA). Results were expressed as mean ± standard deviation (SD) of IOD values for both MMPs across all experimental groups.

### RTqPCR

Total RNA from the lung of post-lactation dams and their female offsprings at PND21 (*n* = 5/group) was extracted using TRIzol Reagent (Thermo Fisher Scientific), according to the manufacturer’s instructions. For mRNA expression analysis, 125 ng/µL aliquots of total RNA were reverse-transcribed in a 16 µL reaction using the High-Capacity RNA-to-cDNA Kit (Life Technologies, 4387406, Carlsbad, CA, USA), following the manufacturer’s protocol. cDNA aliquots from each sample were combined with GoTaq qPCR Master Mix (Promega Corporation, Madison, USA), sense and antisense primers, and the final volume was adjusted to 15 µL with ultrapure water. Primers (stem-loop, forward, and reverse) were synthesized by Biotecnologia (Paulínia, São Paulo, Brazil). Reactions were performed in duplicate in 96-well plates using the QuantStudio 12 K Flex Real-Time PCR System (Applied Biosystems). Relative mRNA expression was calculated using the method described by Livak and Schmittgen (2001) [35]. Expression levels were normalized using the reference genes *Gusb* and *Gapdh*.

### Western blotting

Total protein (70 µg/*n* = 5/per group) from the lung of dams (*n* = 4/group) and female offspring rats (*n* = 5/group) was separated by 10% polyacrylamide gel electrophoresis under reducing conditions (SDS-PAGE). The proteins were then transferred to a nitrocellulose membrane (Amersham, Little Chalfont, UK), blocked with skim milk (5%) for 1 h, and incubated overnight (4 °C) with specific primary antibodies (PI3K, 1:1000, Cell Signaling, 5569 S; AKT 1/2/3, 1:1000, Cell Signaling, #9272; mTOR, 1:1000; Cell Signaling, 2972 S; Aquaporin-1, 1:1000, Abclonal, a15030; ZO-1, 1:1000, Santa Cruz, Sc-8146; PECAM-1, 1:1000, Santa Cruz, sc-1506; B-actin, 1:10000, Sigma, A2228). Subsequently, the membranes were washed in TBS-T and incubated with HRP-conjugated secondary antibody at room temperature for 2 h (anti-Rabbit, Abcam, ab721 1:10000; anti-Mouse, Abcam, ab6789 1:10000; anti-Goat, Abcam, ab7125 1:10000). Reactions were revealed using an ECL kit (Amersham, USA), and band images were captured using a CCD camera (G: BOX system - Syngene, Cambridge, UK). Quantitative band analysis was performed using ImageJ software (National Institutes of Health, USA). The expression levels of the targets were normalized relative to β-actin (endogenous – 42 kDa), and the results were expressed as relative variation, presented as mean ± standard deviation.

### Enzyme assays

Lung oxidative stress parameters were evaluated in post-lactation dams and their female offspring at PND21 (*n* = 5/group). Lung tissues were homogenized, and total protein concentration was determined using the Bradford method. Antioxidant defenses, including superoxide dismutase (SOD) and catalase (CAT) activities, reduced glutathione (GSH) levels, and lipid peroxidation (MDA/TBARS), were assessed using established colorimetric assays. SOD activity was determined by monitoring nitroblue tetrazolium reduction, CAT activity by measuring hydrogen peroxide decomposition, GSH levels using the DTNB method, and lipid peroxidation through quantification of MDA/TBARS. Results were normalized to protein content and expressed according to the respective assay protocols.

### Functional enrichment analysis

The ClusterProfileR package [36–39] was used to perform enrichment analysis, identifying metabolic pathways based on the KEGG database and biological processes using the Gene Ontology Biological Process (GO: BP) database for the key genes analyzed in the mother and post-lactation female groups in PND21. Metabolic pathways and biological processes enriched with a false positive rate (FDR) < 0.05 were considered statistically significant to ensure robustness and minimize false-positive results. The most relevant results, considering the biological context of the genes identified in the mother and post-lactation female groups in PND21, were visualized using a Sankey-Dot plot generated with the SRPlot platform [40], allowing the interpretation of functional differences between the experimental groups.

### Statistical analysis

Statistical analyses were performed using GraphPad Prism^®^ software (version 8.0.1, GraphPad, Inc., San Diego, CA). The Shapiro-Wilk test was used to test the normality of the data. The parametric data were submitted to the *T Student test*, and the non-parametric data were evaluated by the Mann-Whitney test. Data were expressed as mean ± SD. Differences were considered statistically significant when *p* < 0.05.

## Results

### Maternal malnutrition alters biometrical and structural parameters in Dams and female offspring

**Fig. 1:**
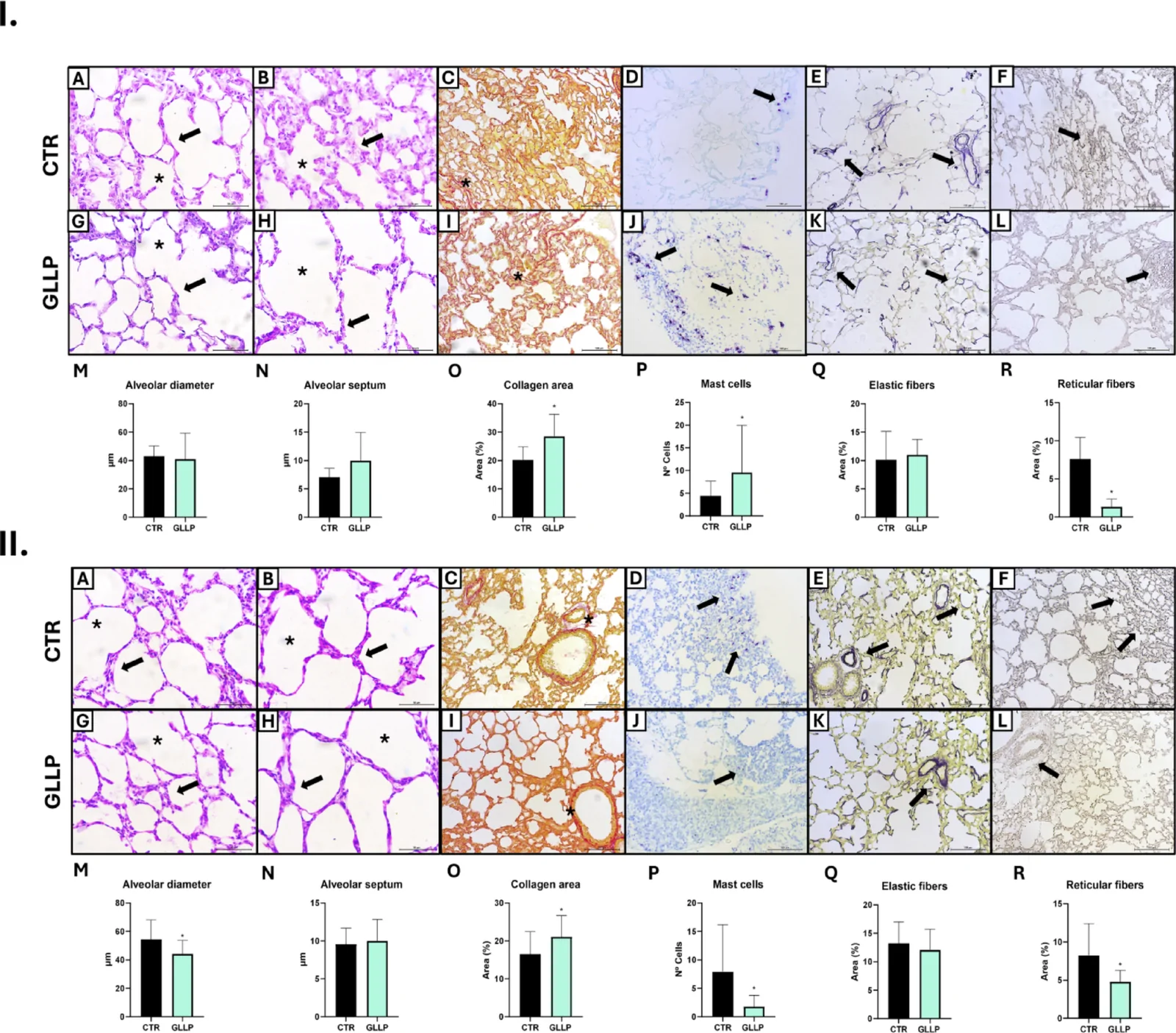
Morphometric analyses of the lungs of post-lactating CTR and GLLP rats. (I) **A** and **G** Hematoxylin-eosin staining in the lungs of CTR and GLLP rats, respectively. **M** Alveolar diameter. **B** and **H** Hematoxylin-eosin staining in the lungs of CTR and GLLP rats, respectively. **N** Alveolar septum. Arrows and asterisk indicate alveolar sacs and septa. **C** and **I** Picrosirius histochemistry in the lungs of CTR and GLLP rats, respectively. Arrows indicate collagen position. **O** Collagen area. **D** and **J** Toluidine blue staining in the lungs of CTR and GLLP rats, respectively. Arrows indicate mast cells. **P** Number of mast cells. **E** and **K** Resorcinol-fuchsin staining in the lungs of CTR and GLLP rats, respectively. Arrows indicate elastic fibers. **Q** Elastic fiber area. **F** and **L** Reticulin staining in the lungs of CTR and GLLP rats, respectively. Arrows indicate reticular fibers. R. Reticular fiber area. (II) Morphometric analyses of the lungs of CTR and GLLP rats in PND21. **A** and **G** Hematoxylin-eosin staining in the lungs of CTR and GLLP rats, respectively. **M** Alveolar diameter. **B** and **H** Hematoxylin-eosin staining in the lungs of CTR and GLLP rats, respectively. **N** Alveolar septum. Arrows and asterisk indicate alveolar sacs and septa. **C** and **I** Picrosirius histochemistry in the lungs of CTR and GLLP rats, respectively. Arrows indicate collagen position. **O** Collagen area. **D** and **J** Toluidine blue staining in the lungs of CTR and GLLP rats, respectively. Arrows indicate mast cells. **P** Number of mast cells. **E** and **K** Resorcinol-fuchsin staining in the lungs of CTR and GLLP rats, respectively. Arrows indicate elastic fibers. **Q** Elastic fiber area. **F** and **L** Reticulin staining in the lungs of CTR and GLLP rats, respectively. Arrows indicate reticular fibers. **R** Reticular fiber area. 20x magnification (collagen area, mast cell count, elastic fibers, and reticular fibers) and 40x magnification (alveolar diameter and alveolar septum) in morphometric analyses of the lungs of post-lactating CTR and GLLP rats. Data expressed as mean ± standard deviation, considered significant when *p* < 0.05 (*)

In the lungs of dams, we observed an increased collagen area and mast cells in the GLLP group (Fig. 1I - C, *I*,* O*,* D*,* J*,* P*), although a decreased reticular fiber (Fig. 1I - F, *L*,* R*) in the GLLP group compared to CTR. In the alveolar diameter, alveolar septum, and elastic fibers (Fig. 1I - A, *G*,* M*,* B*,* H*,* N*,* E*,* K*,* Q*), we did not observe differences in dams. In the female lungs, we observed a significant decrease in the alveolar diameter, mast cells, and reticular fibers in the GLLP group at PND21 when compared to CTR (Fig. 1*II - A*,* G*,* M*,* D*,* J*,* P*,* F*,* L*,* R*), although an increase in the collagen fibers (Fig. 1*II - C*,* I*,* O*). However, we showed no differences in the alveolar septum and elastic fibers (Fig. 1*II - B*,* H*,* N*,* E*,* K*,* Q*).

### MPR does not affect maternal lung tissue remodeling but increases the action of MMP2 in post-weaning females

**Fig. 2:**
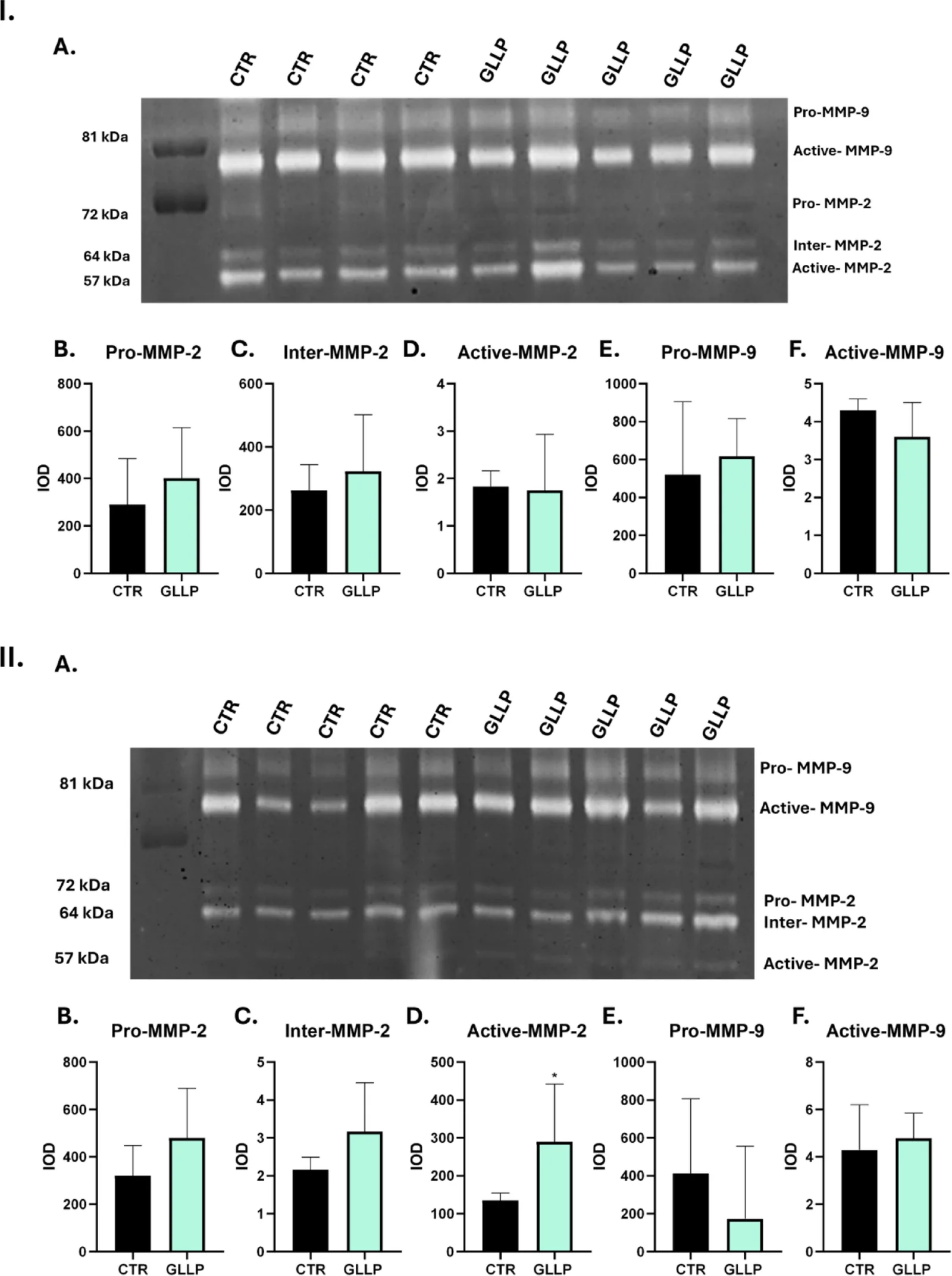
Evaluation of matrix metalloproteinase (MMP) activity in the lungs of post-lactation mothers (I). **A** Representation of the gel and location of MMPs (molecular weight). **B** Pro-MMP-2. **C** Inter-MMP-2. **D** Active MMP-2. **E** Pro-MMP-9 and **F** Active MMP-9. Evaluation of matrix metalloproteinase (MMP) activity in the lungs of female offspring at post-lactation (PND21 - II). **A** Representation of the gel and location of MMPs (molecular weight). **B** Pro-MMP-2. **C** Inter-MMP-2. **D** Active MMP-2. **E** Pro-MMP-9 and **F** Active MMP-9. *CTR* Control group, *GLLP* Gestational and Lactational Low-Protein group, *MMP* Matrix Metalloproteinase. *n* = 5/group. Data normality was assessed using the Shapiro–Wilk test. Parametric data were analyzed using the Student’s *t*-test. Data expressed as mean + standard deviation, considering significant when *p* < 0.05 (*)

Analysis of matrix metalloproteinase (MMP) activity in lung tissue from post-lactation rats showed no significant differences in the pro-, intermediate, or active forms of MMP-2, nor in MMP-9 levels (Fig. 2I *A-F*). In female rats at PND21, active MMP-2 levels increased significantly (Fig. 2I*I D*), whereas no significant changes were detected in pro and intermediate MMP-2 activity, in addition to MMP9 forms (Fig. 2I*I A–F*).

### The PI3K-AKT-mTOR signaling pathway is not modulated in the lungs of mothers exposed to MPR, but shows consistent alterations in females at PND21

**Fig. 3:**
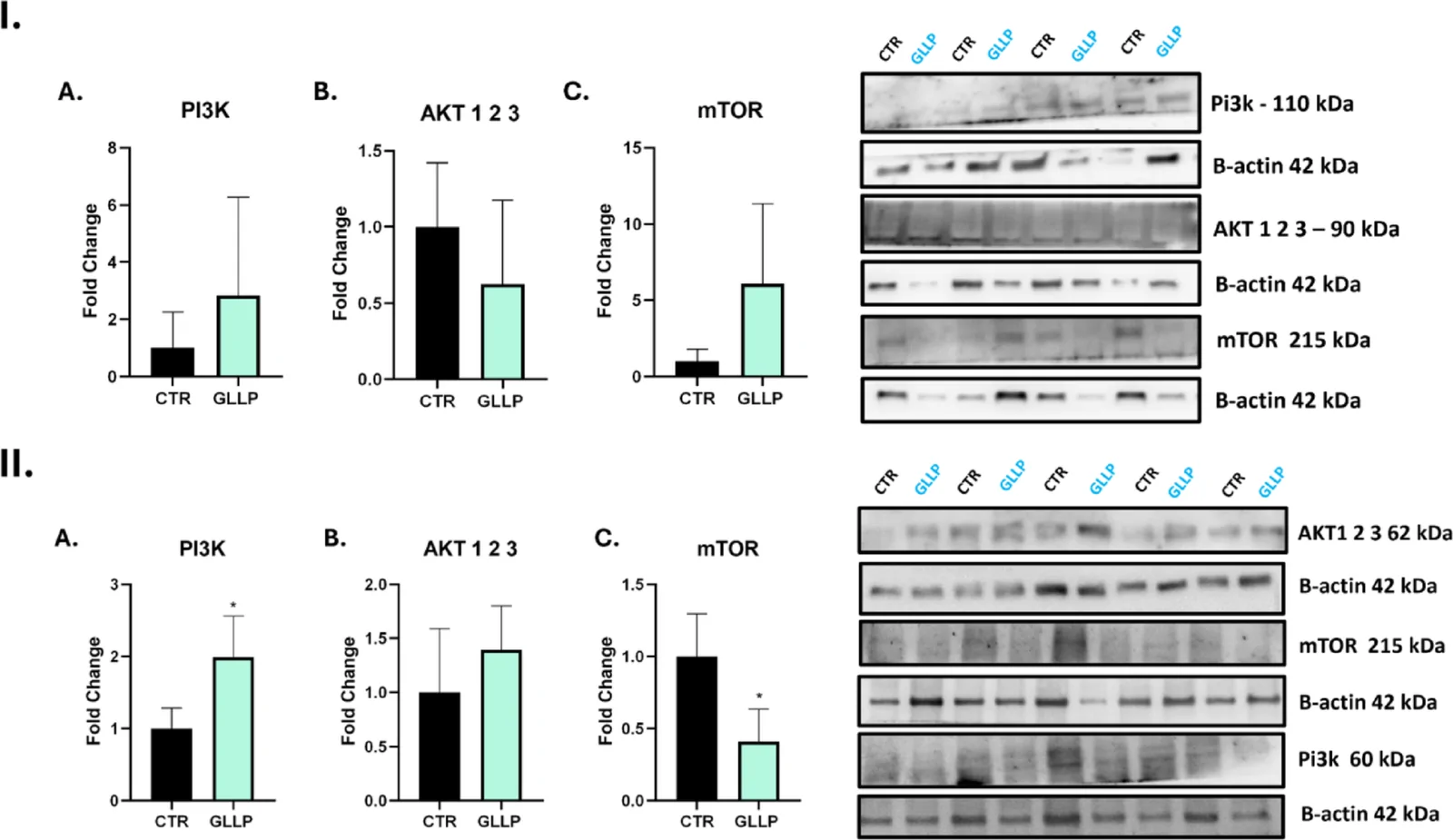
Protein expression by western blotting in the lungs of post-lactation rats (I). **A** Pi3k. **B** AKT 123. **C** mTOR. Data expressed as mean + standard deviation, considering significant when *p* < 0.05 (*). Protein expression by western blotting in the lungs of rats on postnatal day 21 (PND21 - II). **A** Pi3k. **B** AKT 123. **C** mTOR. *CTR* Control group, *GLLP* Gestational and Lactational Low-Protein group, *PI3K* Phosphoinositide 3-kinase, *AKT* Protein kinase B, *mTOR* Mechanistic Target of Rapamycin. *n* = 4–5/group. Data normality was assessed using the Shapiro–Wilk test. Parametric data were analyzed using the Student’s *t*-test. Data expressed as mean + standard deviation, considering significant when *p* < 0.05 (*)

In lung tissue from female dams, no significant differences were observed in the protein expression of PI3K, AKT1/2/3, or mTOR (Fig. 3I). In contrast, in female offspring, PI3K expression was increased and mTOR expression was decreased, while no significant changes were detected for AKT1/2/3 (Fig. 3I*I*).

### MPR does not lead to pulmonary oxidative stress in GLLP mothers and females

**Fig. 4:**
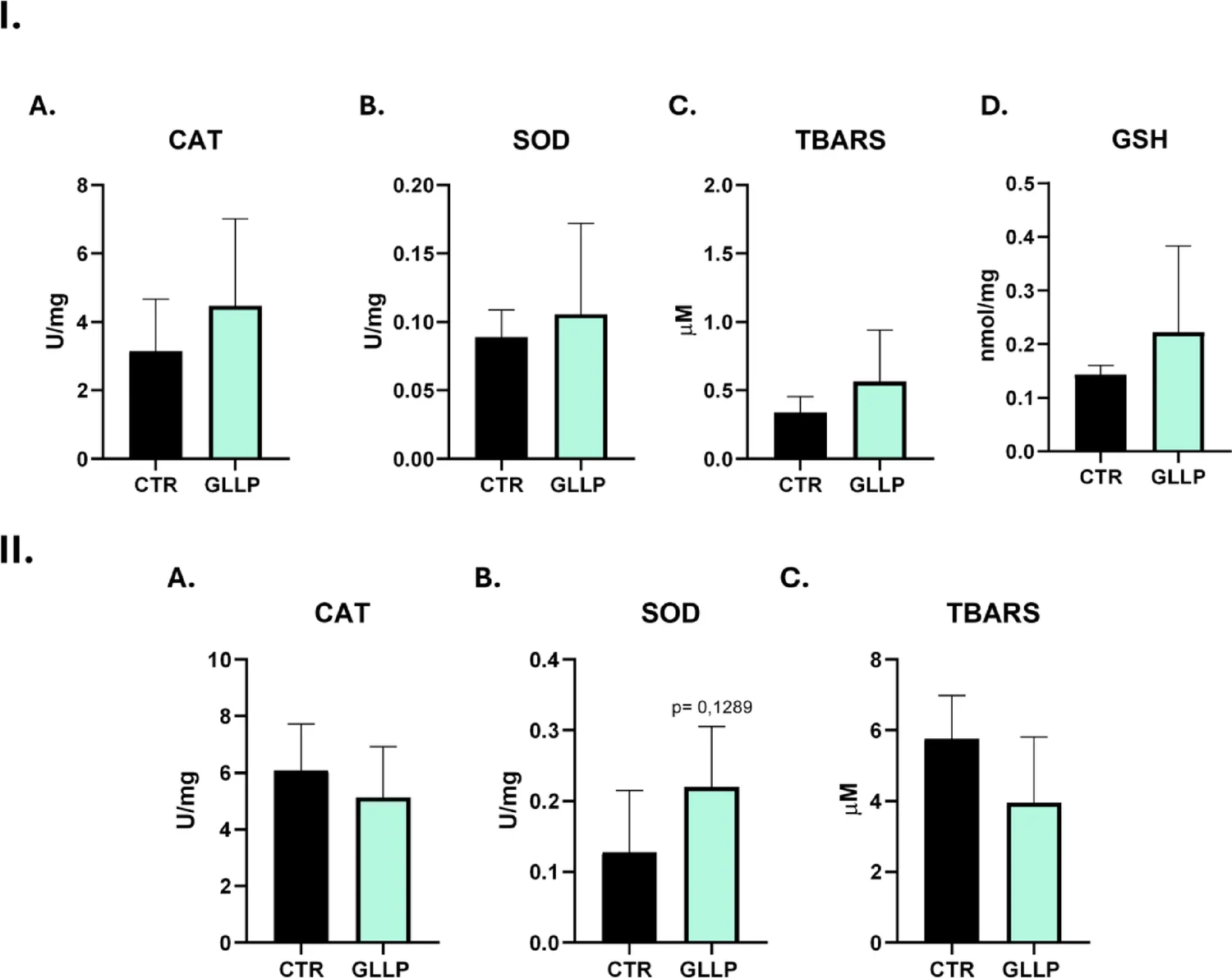
Enzymatic assay to measure antioxidant enzymes in the lungs of dams (I) – **A** CAT. **B** SOD. **C** TBARS. **D** GSH. Data expressed as mean + standard deviation, considering significant when *p* < 0.05 (*). Enzymatic assay to measure antioxidant enzymes in the lungs of female offspring (II) – **A** CAT. **B** SOD. **C** TBARS. *CTR* Control group, *GLLP* Gestational and Lactational Low-Protein group, *CAT* Catalase, *SOD* Superoxide-dismutase, *GSH* Reduced Glutathione, *TBARs* Thiobarbituric Acid Reactive Substances. *n* = 5/group. Data normality was assessed using the Shapiro–Wilk test. Parametric data were analyzed using the Student’s *t*-test. Data expressed as mean + standard deviation, considering significant when *p* < 0.05 (*)

The analysis of antioxidant enzymes from post-lactation mothers showed no significant differences in catalase (CAT), superoxide dismutase (SOD), thiobarbituric acid reactive substances (TBARS), and glutathione (GSH) (Fig. 4I). In female PND21 rats, we observed no significant differences for CAT, SOD, and TBARS (Fig. 4*II – A*,* B and C*).

### At the molecular level, MPR mothers exhibit increased cytoskeletal components, while females show impacts associated with contractile proteins and energy metabolism

**Fig. 5:**
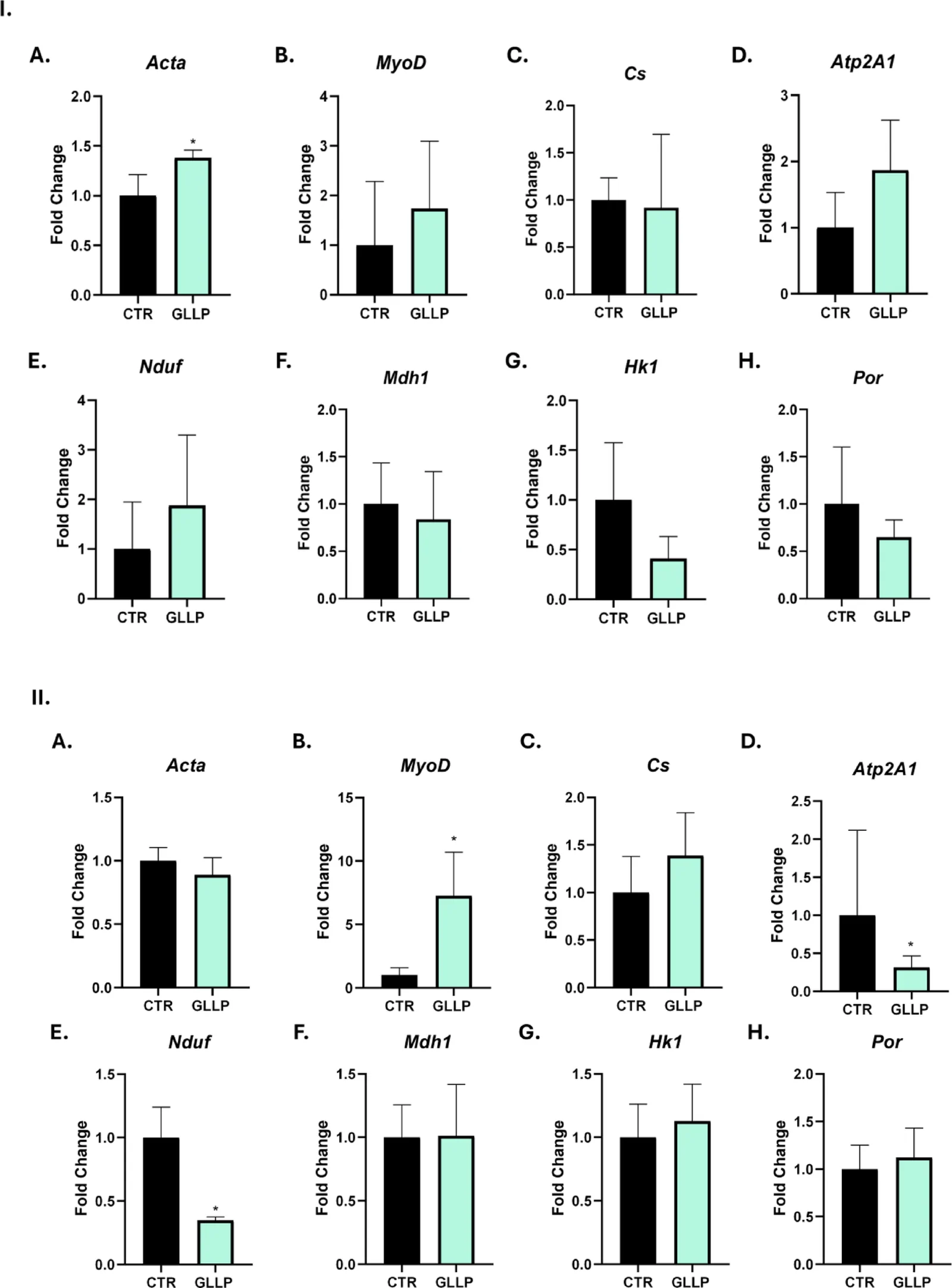
Representative graphs of gene expression in post-lactation rats (I) and PND21 female rats (II). (I) **A ***Acta*. **B ***Myod*. **C** *Cs*. **D** *Atp2a1*. **E** *Nduf*. **F** *Mdh1*. **G** *Hk1*. **H** *Por*. from RT-qPCR analysis with samples evaluated in duplicate. Data are expressed as mean ± SD. * Statistical difference between experimental groups with *p* < 0.05, using Student’s t-test. (II) **A** *Acta.* **B ***Myod*. **C** *Cs*. **D** *Atp2a1*. **E** *Nduf*. **F** *Mdh1*. **G** *Hk1*. **H** *Por*. from RT-qPCR analysis with samples evaluated in duplicate. *CTR* Control group, *GLLP* Gestational and Lactational Low-Protein group, *Acta* Actin Alpha, *Myod* Myogenic Differentiation, *Cs* Citrate synthase, *Atp2a1* ATPase sarcoplasmic/endoplasmic reticulum Ca2+²⁺ transporting 1, *Nduf* NADH Dehydrogenase Ubiquinone Factor, *Mdh1* Malate Dehydrogenase 1, *Hk1* Hexokinase 1, *Por* Cytochrome P450 oxidoreductase. *n* = 5/group. Data normality was assessed using the Shapiro–Wilk test. Parametric data were analyzed using the Student’s *t*-test. Data are expressed as mean ± SD. * Statistical difference between experimental groups with *p* < 0.05, using Student’s t-test

Molecular gene expression analyses of post-lactation mothers revealed that an MPR gene expression module is related to muscle differentiation and energy metabolism. A significant increase in *Acta* expression was observed in the GLLP group (Fig. 5*I - A*). Furthermore, no statistically significant differences were found for *MyoD*, *Cs*, *Atp2A1*, *Nduf*, *Mdh1*, *Hk1*, and *Por* (Fig. 5*I - B*,* C*,* D*,* E*,* F*,* G*,* and H*).

In PND21 females, assessment of gene expression by RT–qPCR demonstrated a significant increase in Myod expression in the GLLP group. In contrast, a significant reduction in *Atp2A1* and *Nduf* expression was observed in treated females (Fig. 5*II - B*,* D*,* and E*). No statistically significant differences were observed in *Acta*, *Cs*, *Mdh1*, *Hk1*, and *Por* (Fig. 5*II - A*,* C*,* F*,* G*,* and H*).

### In silico analyses indicate that MPR alters the pulmonary structure of both mothers and post-weaning female offspring, affecting cell signaling pathways and biological responses in females

**Fig. 6:**
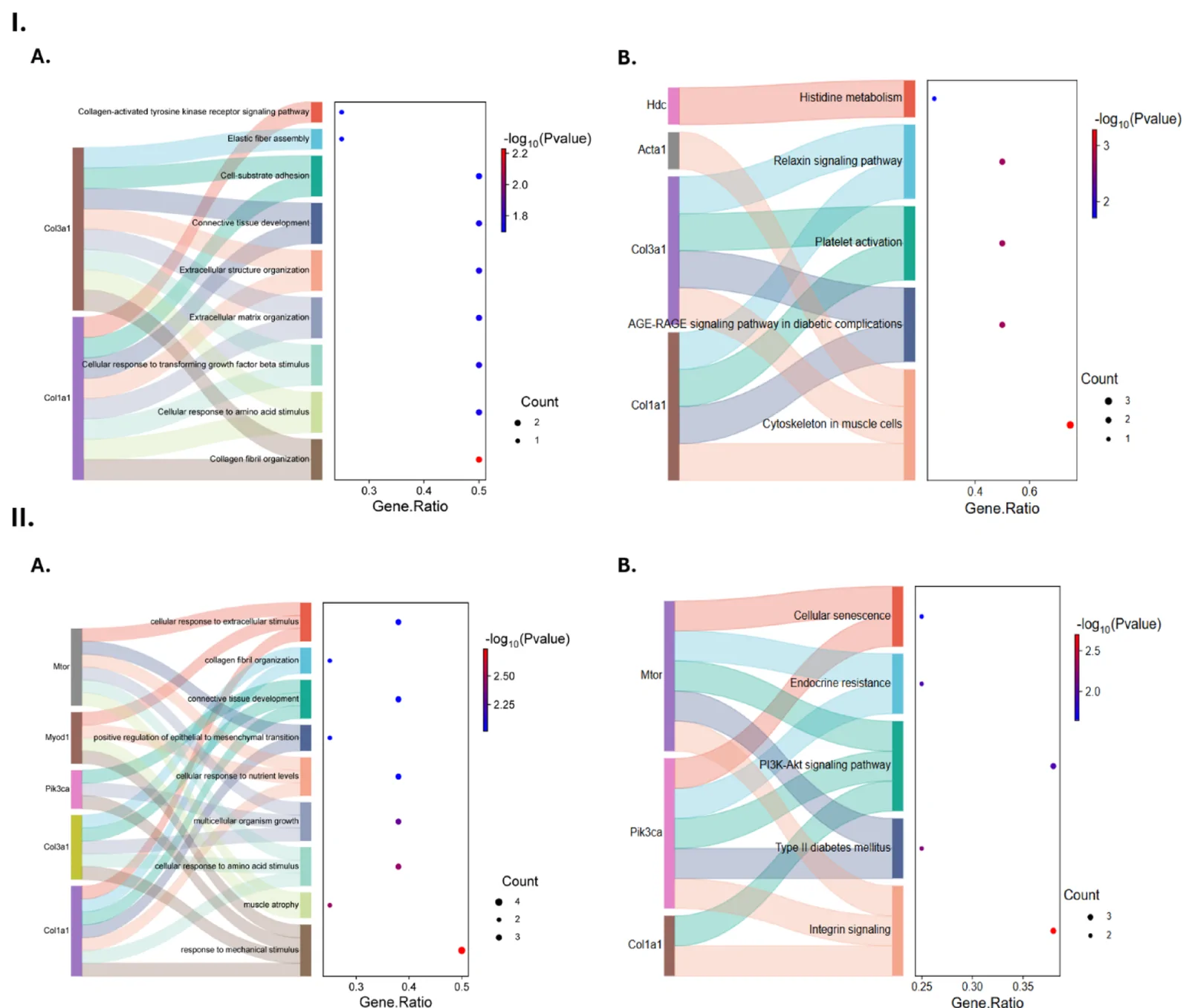
Functional enrichment analyses and pathway interactions of differentially expressed genes associated with alterations observed in maternal lungs (I) and post-weaning female lungs (II). Integrated enrichment analyses are presented using Sankey diagrams and bubble diagrams, illustrating the relationships between genes, biological processes, and signaling pathways associated with deregulated enriched targets investigated in the lungs of mothers (I - **A**, **B**) and female offspring (II - **A**, **B**). Prominent pathways include PI3K-Akt, MAPK, AMPK, HIF-1 signaling, apoptosis, cellular senescence, and metabolic pathways, indicating coordinated regulation of cellular stress responses, energy metabolism, and structural remodeling. Corresponding bubble diagrams display the enriched pathways ranked by gene ratio and statistical significance (− log₁₀ p-value), with bubble size representing gene count. CTR. Control group. GLLP. Gestational and Lactational Low-Protein group. *Acta* Actin Alpha, *Myod* Myogenic Differentiation, *Cs* Citrate synthase, *Atp2a1* ATPase sarcoplasmic/endoplasmic reticulum Ca2+²⁺ transporting 1, *Nduf*. NADH Dehydrogenase Ubiquinone Factor, *Mdh1* Malate Dehydrogenase 1, *Hk1* Hexokinase 1, *Por* Cytochrome P450 oxidoreductase, *CAT* Catalase, *SOD* Superoxide-dismutase, *GSH* Reduced Glutathione, *PI3K* Phosphoinositide 3-kinase, *AKT* Protein kinase B, *mTOR* Mechanistic Target of Rapamycin. *COL1A1* Collagen Type I Alpha 1 Chain, *COL3A1* Collagen Type III Alpha 1, *MMP* Matrix Metalloproteinase

In the in silico analysis of the dams, in the analysis focused on differentially expressed genes (Figs. 6*I - A and B*), significant alterations were identified, mainly associated with structural components of lung tissue. Enrichment was observed in processes such as extracellular matrix organization, connective tissue development, collagen fiber formation, and response to TGF-β, with emphasis on the *Col1a1* and *Col3a1* genes. Additionally, the KEGG pathway analysis revealed significant involvement of pathways such as platelet activation and cytoskeleton organization.

In the in silico analysis of young females, the analysis focused on differentially expressed genes (Fig. 6*II - A and B*) revealed significant alterations in processes associated with tissue structure and adaptation. Enrichments were observed in pathways related to collagen organization, connective tissue development, and epithelial-mesenchymal transition.

## Discussion

The major innovation of this study is the demonstration that MPR affects pulmonary health in both dams and female offspring, providing a generational perspective with direct relevance to women’s health within the DOHaD framework. By integrating morphological, molecular, biochemical, and in silico approaches, we demonstrate that MPR induces structural remodeling in maternal lungs, potentially impairing ventilatory function. Furthermore, female offspring exhibited alterations consistent with delayed pulmonary development, including extracellular matrix remodeling, changes in smooth muscle-related pathways, and disturbances in energy metabolism and growth-associated signaling pathways. These findings address a critical gap in the literature by expanding the investigation of MPR beyond offspring and into maternal health, while also highlighting female-specific pulmonary responses compared with previous studies predominantly focused on males. Collectively, this work provides new insights into the developmental consequences of maternal malnutrition and identifies potential mechanisms linking early-life nutritional adversity to the developmental origins of respiratory disorders.

The effects of maternal diet can be significant for homeostasis or imbalances in lung morphophysiology, and our results demonstrated disturbances in both dams and the female structure lungs. Epidemiological data analysis demonstrated that preterm neonates with low weight for gestational age are more likely to have chronic respiratory disorders [41, 42], which is associated with maternal diet [14, 43–44]. Children who are born at term with intrauterine growth restriction can have negative pulmonary sequelae such as bronchopulmonary dysplasia [45], and are more susceptible to respiratory diseases during the development phase [46]. Respiratory physiology might be disturbed, and this condition can contribute to the development of asthma [14, 15, 47–48]. Recent research has revealed that MPR impacts collagen deposition in the lungs, inflammatory cell recruitment, oxidative damage, and antioxidant defense in animal models [49]. All these conditions can lead to structural change in the lungs, with several consequences to respiratory function.

Dams showed lung remodeling with increased collagen and mast cells and reduced reticular fibers. Female offspring had similar matrix changes but with fewer mast cells and smaller alveoli. Interestingly, in males, MPR reduced alveolar diameter without matrix changes, but induced tight junction disruption and age-related fibrotic and inflammatory lung alterations [27]. The impacts on collagen I and III deposition may correlate with airway remodeling, and studies have evaluated reticular basement membrane thickening and pro-fibrotic airway profile as a potential risk for the development of asthma [50]. Excessive collagen deposition in alveolar areas is associated with lung fibrosis [51]. Experimental studies with 12-week-old C57BL/6 mice associated an increased Col 1/Col 3 ratio with idiopathic pulmonary fibrosis [52]. Other experimental models with antenatal exposure to endotoxin have demonstrated changes in the airways and distal lung, such as decreased alveolar surface area, as a risk factor for bronchopulmonary dysplasia [53].

Previously, Barata et al. (2025) demonstrated that MPR induces steatohepatitis and adrenal alterations in dams at the end of lactation, indicating significant metabolic adjustments [54]. The results demonstrate distinct pulmonary responses between post-lactation mothers and PND21 females. In mothers, increased collagen and mast cells, along with reduced reticular fibers, indicate inflammatory and fibrotic remodeling [55, 56], while alveolar structure remained preserved. In contrast, female offspring showed reduced alveolar diameter, suggesting delayed alveolarization [57], decreased mast cells indicating altered immune response [55], and increased collagen with reduced reticular fibers, consistent with early extracellular matrix remodeling [58]. Alveolar structure was preserved, but MPR induced remodeling in adult lungs and impaired alveolar development in offspring, highlighting increased vulnerability of the immature lung [56]. In line with these findings and supported by in silico pathway analysis, our results suggest that maternal lungs exhibit a phenotype prone to pro-fibrotic and asthmatic conditions, likely reflecting compensatory and adaptive responses to metabolic stress during lactation, particularly under severe protein restriction. In contrast, female offspring display features consistent with delayed lung development, including reduced gas-exchange structures, accompanied by signs of immune system attenuation.

Matrix metalloproteinases (MMPs) constitute a family of zinc-dependent endopeptidases responsible for the degradation and remodeling of the extracellular matrix (ECM), playing essential roles in various physiological processes [59, 60]. Among them, MMP-9 is critically involved in lung remodeling, ECM reorganization, alveolar development, and inflammatory responses [61]. The reduction in MMP-9 observed in female offspring in the present study may be associated with delayed lung development and reduced tissue plasticity, potentially resulting from the nutritional conditions experienced during gestation and lactation [62]. Previous studies have predominantly investigated these effects in males, demonstrating that MPR increases MMP-9 expression. In contrast, findings in females suggest a sex-specific response, possibly mediated by estrogen, which has been shown to negatively regulate MMP-9 expression in tissues such as the uterus and lungs [63]. Moreover, the significant alterations in MMP-2 isoforms—enzymes primarily involved in structural and homeostatic ECM remodeling—indicate that essential tissue maintenance was deregulated following early-life MPR exposure [64]. The differential modulation of MMP-2, but not MMP-9, suggests selective activation of pathways related to early tissue stress or injury rather than broad structural remodeling, potentially reflecting endocrine resistance and adaptive responses to nutritional stress and developmental cues, as indicated by pathway enrichment analysis.

At PND21 female lungs, PI3K expression was increased while mTOR was reduced, with no changes in AKT1/2/3, aquaporin-1, PECAM-1, ZO-1, or HIF-1. This pattern suggests selective modulation of the PI3K/AKT/mTOR pathway, with elevated PI3K but no downstream activation, indicating possible functional inhibition as an adaptive response to MPR and limited amino acid availability [65, 66]. The reduction in mTOR further supports metabolic adjustment during a critical nutritional window [67]. The stability of AKT and other markers indicates preserved epithelial, endothelial, and hypoxia-related functions, suggesting maintenance of pulmonary structural homeostasis in early life. Protective effects of female sex hormones may also contribute to this stability [68, 69]. Additionally, the absence of changes in dams supports a developmental origin of these alterations. Overall, MPR appears to induce early intracellular signaling changes in female lungs without overt structural impairment at this stage. Interestingly, the increase in PI3K expression, accompanied by a reduction in mTOR levels and maintenance of AKT, may suggest a response to growth or insulinemic factors; however, with a dissociation of the canonical PI3K/AKT/mTOR pathway in post-weaning female lung cells, a mechanism that can be interpreted as adaptive to the effects of MPR. Specifically, it is plausible to consider that early in life, given the conditions, lung cells prioritize survival signaling over anabolism and growth, conditions that may accurately reflect the parameters of developmental delay and structural and metabolic impacts corroborated through histological and molecular analyses.

Analysis of antioxidant enzymes showed that MPR did not significantly alter pulmonary redox status in either post-lactation dams or PND21 female offspring. In dams, CAT, SOD, TBARS, and GSH levels were unchanged, indicating preserved redox homeostasis. Similarly, females showed no significant changes in CAT, SOD, or TBARS, although a trend toward increased SOD activity and possible H₂O₂ production was observed. These findings suggest maintenance of antioxidant capacity and structural integrity, likely through compensatory mechanisms. While studies in male models report increased oxidative stress after MPR, with elevated TBARS and altered antioxidant defenses [49], responses appear to be sex-specific, with females exhibiting greater adaptive resilience [70]. Overall, female offspring display preserved antioxidant defenses and no detectable pulmonary oxidative damage during lactation and early in life.

Gene expression analyses in post-lactation dams revealed selective alterations, notably increased *Acta* expression in the GLLP group, suggesting structural adaptation of pulmonary muscle tissue related to cytoskeletal organization and contractility [71]. In contrast, the absence of changes in *MyoD* indicates that primary myogenic regulatory pathways were not affected, pointing to modulation of structural components rather than early differentiation processes [72, 73]. Markers of mitochondrial and metabolic function, including *Cs* and *Nduf*, as well as glycolytic (*Hk1*) and intermediary metabolism (*Mdh1*) genes, remained unchanged, indicating preserved energy metabolism and oxidative capacity. Similarly, Atp2A1 and Por expression stability suggests maintenance of calcium handling and redox balance [74, 75]. Overall, these findings indicate that MPR induces selective gene expression changes, primarily affecting structural muscle components while preserving core metabolic pathways, consistent with adaptive responses to nutritional stress and developmental programming.

Gene expression analysis in PND21 female offspring indicates overall preservation of the metabolic profile following MPR, with no significant changes in *Acta*, *Cs*, *Atp2A1*, *Mdh1*, *Hk1*, or *Por*, suggesting maintained structural, mitochondrial, and glycolytic pathways. However, *MyoD* expression was significantly increased in the GLLP group, pointing to early activation of myogenic differentiation as a potential adaptive response to adverse intrauterine conditions [72]. In contrast, reduced *Nduf* expression suggests subtle impairment of mitochondrial electron transport chain function, potentially reflecting decreased oxidative phosphorylation efficiency [76]. Collectively, these findings indicate that MPR induces selective gene expression changes in female offspring, affecting differentiation and mitochondrial function despite overall metabolic stability, consistent with adaptive responses (atrophy response and cellular response to nutrient levels) highlighted in in silico pathway enrichment analyses.

Interestingly, several studies have demonstrated that maternal malnutrition, including MPR, alters glucocorticoid programming in offspring [77]. Glucocorticoids are known to play important roles in the regulation of sex-specific developmental responses [78], and the effects of MPR on fetal development may be mediated, at least in part, by reduced placental 11β-hydroxysteroid dehydrogenase (11β-HSD) activity, which regulates fetal exposure to maternal glucocorticoids [79]. These mechanisms are particularly relevant to pulmonary development, as the physiological increase in maternal glucocorticoids during late gestation is essential for the maturation of type II pneumocytes and surfactant production. Previous studies have reported increased glucocorticoid receptor (GR) mRNA and protein expression in peripheral tissues, including the kidneys and lungs, both in late-gestation fetuses and in offspring up to 12 weeks of age following MPR [77, 80, 81]. These findings suggest that inadequate maternal nutrition may enhance glucocorticoid sensitivity in offspring, thereby increasing susceptibility to metabolic disorders later in life [77, 80, 81]. Importantly, glucocorticoid responses exhibit marked sexual dimorphism. Under physiological conditions, females generally display greater hypothalamic-pituitary-adrenal axis responsiveness than males, and maternal stressors during pregnancy may differentially influence developmental programming in male and female offspring [82, 83]. However, the consequences of endogenous glucocorticoid exposure induced by MPR are likely distinct from those resulting from exogenous glucocorticoid administration. MPR represents a complex nutritional stressor that induces multiple physiological alterations, including changes in placental function, fetal nutrient availability, oxidative stress, inflammation, endocrine signaling, and glucocorticoid metabolism. Thus, developmental programming in this model reflects the integrated effects of several interacting pathways rather than glucocorticoid signaling alone. For example, MPR-induced hypertension has been reported to be glucocorticoid-dependent in males but not in females, and MPR also impairs fetal renal development by increasing apoptosis and reducing nephrogenesis, changes that may contribute to adult renal dysfunction and hypertension [84]. Accordingly, sex-specific differences observed following MPR likely result from the interaction between glucocorticoids and other nutritional, metabolic, placental, and epigenetic mechanisms. Therefore, available evidence suggests that pulmonary alterations are more pronounced in post-weaning males than in females, underscoring the importance of considering sexual dimorphism and the underlying biological context when investigating the developmental origins of respiratory disorders.

Importantly, these sex-specific pulmonary responses may be closely associated with differences in sex steroid signaling, anabolic regulation, circadian rhythmicity, corticosterone dynamics, epigenetic programming, and sex-specific placental gene expression. Because sexual divergence is established early during development, these factors can differentially shape developmental trajectories and physiological adaptations, with sex-dependent responses to early-life nutritional insults emerging during fetal life and becoming more pronounced after puberty, when sex hormone production further accentuates biological differences. Consistent with this concept, although both sexes exhibited reduced alveolar diameter at PND21, females showed additional decreases in reticular fibers, mast cell density, and active MMP-9, together with increased collagen deposition, whereas males displayed no changes in collagen content or mast cell number and instead exhibited increased MMP-2 isoforms [27]. These findings indicate sex-specific differences in extracellular matrix remodeling and immune responses during early lung development. Moreover, pulmonary signaling also differed between the sexes: females exhibited increased PI3K and reduced mTOR levels, whereas males showed increased AKT and mTOR expression [27], highlighting distinct molecular pathways underlying pulmonary adaptation to MPR.

From a global perspective and within the framework of the DOHaD, although MPR induces steatohepatitis and profound molecular alterations in the adrenal glands of dams at the end of lactation [54], our findings indicate that the physiological adaptations associated with pregnancy and lactation appear to preserve pulmonary homeostasis, even under conditions of maternal malnutrition. In contrast, female offspring exhibited a clear phenotype of impaired lung development, characterized by alterations in alveolar diameter, smooth muscle-related components (*Myod*), extracellular matrix organization (collagen and reticular fibers, and mast cells), tissue remodeling pathways (MMP2), survival and growth signaling cascades (PI3K and mTOR), and energy metabolism (NDUF). These findings suggest that embryofetal and early postnatal nutritional challenges may induce long-lasting epigenetic reprogramming. Notably, DNA methylation patterns are highly tissue- and cell-specific, supporting the possibility that distinct pulmonary cell populations are differentially affected by early-life nutritional insults. Indeed, several studies have demonstrated epigenetic alterations in pulmonary cells from asthmatic patients, regulating airway remodeling, phagocytosis, and other lung functions [85]. Furthermore, epigenetic modifications within the Il33 promoter have been identified in alveolar type II (AT2) cells, indicating that persistent epithelial alterations following infection may depend on IL-1β-mediated pathways, thereby contributing to asthma susceptibility [86]. In parallel, accumulating evidence suggests that early-life events influencing immune maturation along the gut–lung axis represent a critical window for asthma prevention and are strongly linked to epigenetic regulation [87]. Therefore, from a DOHaD perspective, our study highlights sex-specific pulmonary responses to MPR during early life. Compared with the current literature, we provide evidence that female offspring are particularly susceptible to structural and molecular alterations in the lung. These changes likely reflect underlying epigenetic mechanisms and warrant further longitudinal investigation to identify potential regulatory targets, including DNA methylation, histone modifications (acetylation, methylation, and phosphorylation), and non-coding RNAs. Understanding these mechanisms may clarify how postnatal environmental exposures interact with developmental programming to influence susceptibility to respiratory diseases during adulthood and aging.

Key limitations of this study include the absence of functional assessments to determine whether the observed structural and molecular alterations in mothers and post-weaning females translate into changes in ventilatory, inspiratory, expiratory, and residual capacities. Another important limitation of the study was that it investigated the impacts on some signaling pathways without evidencing these impacts on phosphorylated/active proteins. In addition, systemic parameters relevant to pulmonary responses (e.g., insulin, adrenaline, corticosterone) were not evaluated. Another important limitation was the inability to assess GSH levels in females due to insufficient sample size and methodological variability. Another limitation is that we did not include a non-pregnant, non-lactating control group, making it not possible to distinguish the effects of pregnancy/lactation itself from those of protein restriction. Another limitation is that we did not include a non-pregnant, non-lactating control group, making it not possible to distinguish the effects of pregnancy/lactation itself from those of protein restriction. As a critical limitation, we did not evaluate transgenerational and multigenerational effects; however, given our results, MPR may lead to epigenetic marks in the lungs and extrapulmonary organs, in addition to the metabolic response, which may influence future generations – this is quite plausible when we consider reproductive and ovarian aspects, since ovarian reserve is already established in utero, and therefore we propose mechanistic hypotheses for future investigations on these pulmonary assessments in other generations. Despite these constraints, this study is original in demonstrating the effects of MPR on pulmonary outcomes in both mothers and female offspring, providing relevant insights into female health within the context of global hunger and the Developmental Origins of Health and Disease perspective of respiratory disease development.

## Conclusion

Integrating these findings with the in silico analyses, we demonstrate that MPR induces structural remodeling in maternal lungs, with potential consequences for pulmonary health. In female offspring, MPR was associated with delayed lung development, extracellular matrix remodeling, and adaptive changes in myosin-related pathways, accompanied by reduced energy metabolism markers. Collectively, these findings indicate that MPR adversely affects pulmonary structure and molecular homeostasis in both mothers and offspring early in life, establishing biological alterations that may contribute to the developmental origins of respiratory disorders. Importantly, this study provides a foundation for future investigations assessing long-term pulmonary outcomes, including adult and aged lung function, thereby supporting translational approaches and the clinical validation of developmental programming mechanisms in respiratory disease.

## Supplementary Information

Below is the link to the electronic supplementary material.

Supplementary Material 1.

## Data Availability

No datasets were generated or analysed during the current study.
