# Supplementary material for: Embryo-fetal and early-life protein restriction programs pulmonary structure and molecular parameters in both dams and female offspring
Source: Mol Cell Biochem. 2026 Jul 24;481(9):4303–19. doi: 10.1007/s11010-026-05653-4 (PMC13638724; doi:10.1007/s11010-026-05653-4)
Supplement: Supplementary file 1 [file 11010_2026_5653_MOESM1_ESM.docx]

**Supplementary Files**

Supplementary Figure 1. Experimental design. Sprague Dawley rats were exposed to a normoproteic diet (17% protein - CTR black bar) or a hypoproteic diet (6% protein - GLLP blue bar) during gestation and lactation. Mothers (CTR and GLLP) and female offspring were euthanized at the end of lactation (mothers) and post-weaning for females. GD. Gestational day. PND. Postnatal day.
